# 2-(2-Hydroxy­benzyl­ideneamino)benzonitrile

**DOI:** 10.1107/S160053680801163X

**Published:** 2008-05-10

**Authors:** Rong Xia, Hai-Jun Xu, Xing-Xuan Gong

**Affiliations:** a Ordered Matter Science Research Center, College of Chemistry and Chemical, Engineering, Southeast University, Nanjing 210096, People’s Republic of China

## Abstract

The mol­ecule of the title compound, C_14_H_10_N_2_O, displays a *trans* configuration with respect to the C=N double bond. The mol­ecule is roughly planar; the two aromatic rings make a dihedral angle of 9.3 (3)°. Such a planar conformation is induced by the strong intra­molecular O—H⋯N hydrogen bond between the imine and hydroxyl groups.

## Related literature

For the structures of similar Schiff base compounds, see: Cheng *et al.* (2005 ▶, 2006 ▶). For related literature, see: Chen *et al.* (2008 ▶); Elmah *et al.* (1999 ▶); May *et al.* (2004 ▶); Weber *et al.* (2007 ▶); Xu *et al.* (2008 ▶). For bond-length data, see: Allen *et al.* (1987 ▶).
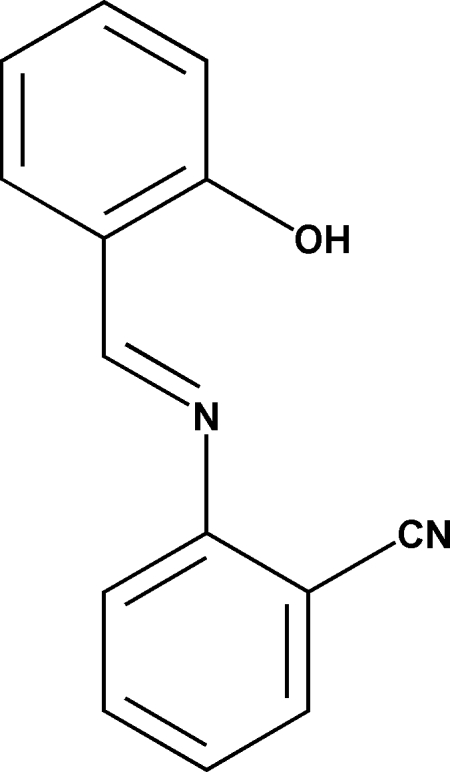


## Experimental

### 

#### Crystal data

C_14_H_10_N_2_O
*M* *_r_* = 222.24
Monoclinic, 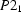

*a* = 4.7667 (10) Å
*b* = 16.190 (3) Å
*c* = 7.6714 (15) Å
β = 93.30 (3)°
*V* = 591.0 (2) Å^3^
*Z* = 2
Mo *K*α radiation
μ = 0.08 mm^−1^
*T* = 293 (2) K
0.20 × 0.05 × 0.05 mm

#### Data collection

Rigaku Mercury2 diffractometer
Absorption correction: multi-scan (*CrystalClear*; Rigaku, 2005 ▶) *T*_min_ = 0.981, *T*_max_ = 1.00 (expected range = 0.977–0.996)
5470 measured reflections
1201 independent reflections
633 reflections with *I* > 2σ(*I*)
*R*_int_ = 0.105

#### Refinement

*R*[*F* ^2^ > 2σ(*F* ^2^)] = 0.061
*wR*(*F* ^2^) = 0.136
*S* = 1.03
1201 reflections
155 parameters
1 restraint
H-atom parameters constrained
Δρ_max_ = 0.14 e Å^−3^
Δρ_min_ = −0.18 e Å^−3^

### 

Data collection: *CrystalClear* (Rigaku, 2005 ▶); cell refinement: *CrystalClear*; data reduction: *CrystalClear*; program(s) used to solve structure: *SHELXS97* (Sheldrick, 2008 ▶); program(s) used to refine structure: *SHELXL97* (Sheldrick, 2008 ▶); molecular graphics: *ORTEPIII* (Burnett & Johnson, 1996 ▶) and *ORTEP-3 for Windows* (Farrugia, 1997 ▶); software used to prepare material for publication: *SHELXL97*.

## Supplementary Material

Crystal structure: contains datablocks I, global. DOI: 10.1107/S160053680801163X/dn2331sup1.cif

Structure factors: contains datablocks I. DOI: 10.1107/S160053680801163X/dn2331Isup2.hkl

Additional supplementary materials:  crystallographic information; 3D view; checkCIF report

## Figures and Tables

**Table 1:** Hydrogen-bond geometry (Å, °)

*D*—H⋯*A*	*D*—H	H⋯*A*	*D*⋯*A*	*D*—H⋯*A*
O1—H1⋯N1	0.82	1.92	2.651 (6)	147

**Fig. 1.**
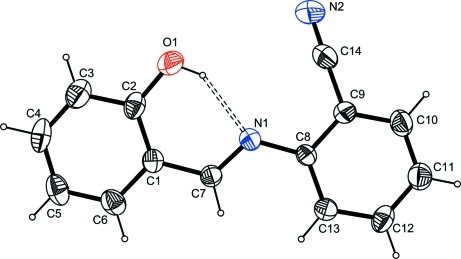
A view of the title compound with the atom-labelling scheme. Displacement ellipsoids are drawn at the 30% probability level. H atoms are represented as smal spheres of arbitrary radii. Intramolecular H bond is shown as dashed line.

## supplementary crystallographic information

### Comment

The Schiff base compounds have received considerable attention for several decades, primarily due to their importance in the development of coordination chemistry related to magnetism (Weber, *et al.*, 2007), catalysis (Chen, *et al.*, 2008) and biological process (May, *et al.*,2004). Recently, we have reported a Schiff base compound (Xu, *et al.*, 2008). As an extention of our work on the structural characterization of Schiff base compounds, the title compound, (I), has been synthesized and its crystal structure is reported here.

As expected, the molecule displays a *trans* configuration about the central C7=N1 bond. The dihedral angle between the planes of the two aromatic rings is 9.34(0.29)°, showing that the conjugated part of the molecule is not entirely coplanar. A strong O – H ··· N intramolecular hydrogen-bond interaction is observed in the molecular structure (Fig. 1, Table 1) similar to the pervious reports (Xu *et al., *2008; Cheng *et al.*,2006, 2005).

All the bond lengths and bond angles in the compound are within normal ranges (Allen, *et al.*, 1987). The C7=N1 bond length of 1.292 (5) Å indicates a high degree of double-bond character comparable with the corresponding bond lengths in other Schiff bases (1.280 (2) Å; Elmah *et al.*, 1999).

### Experimental

All chemicals were obtained from commercial sources and used without further purification except for salicylaldehyde which is distiled under reduced pressure before use. 3-aminobenzonitrile (1.18 g, 10 mmol) and salicylaldehyde (1.22 g, 10 mmol) were dissolved in ethanol (20 ml). The mixture was heated to reflux for 4 h, then cooled to room temperature overnight and large amounts of a yellow precipitate were formed. Yellow crystal was obtained by recrystallization from ethyl alcohol(yield: 85%). ^1^H-NMR(CDCl_3_, 300 MHz): δ6.98 (t, 1 H), 7.08 (d, 1 H), 7.37(t, 2 H), 7.45 (t, 2 H), 7.69 (m, 2H), 8.72 (s, 1 H). Esi-MS: calcd for C_14_H_9_N_2_O – H *m*/*z *221.24, found 221.34. For the X-ray diffraction analysis, suitable single crystals of compound (I) were obtained after one week by slow evaporation from an ethyl alcohol solution.

### Refinement

All H atoms attached to C atoms and O atom were fixed geometrically and treated as riding with C—H = 0.93 Å and O—H = 0.82Å with U_iso_(H) = 1.2U_eq_(C) or U_iso_(H) = 1.5U_eq_(O).

In the absence of significant anomalous scattering, the absolute structure could not be reliably determined and then the Friedel pairs were merged and any references to the Flack parameter were removed.

### Crystal data

**Table:**

C_14_H_10_N_2_O	*F*_000_ = 232
*M**_r_* = 222.24	*D*_x_ = 1.249 Mg m^−^^3^
Monoclinic, *P*2_1_	Mo *K*α radiation λ = 0.71073 Å
Hall symbol: P 2yb	Cell parameters from 4123 reflections
*a* = 4.7667 (10) Å	θ = 3.7–28.7º
*b* = 16.190 (3) Å	µ = 0.08 mm^−^^1^
*c* = 7.6714 (15) Å	*T* = 293 (2) K
β = 93.30 (3)º	Block, colorless
*V* = 591.0 (2) Å^3^	0.20 × 0.05 × 0.05 mm
*Z* = 2	

### Data collection

**Table:**

Rigaku Mercury2 diffractometer	1201 independent reflections
Radiation source: fine-focus sealed tube	633 reflections with *I* > 2σ(*I*)
Monochromator: graphite	*R*_int_ = 0.105
Detector resolution: 13.6612 pixels mm^-1^	θ_max_ = 26.0º
*T* = 293(2) K	θ_min_ = 3.7º
ω scans	*h* = −5→5
Absorption correction: multi-scan(CrystalClear; Rigaku, 2005)	*k* = −19→19
*T*_min_ = 0.981, *T*_max_ = 1.00	*l* = −9→9
5470 measured reflections	

### Refinement

**Table:**

Refinement on *F*^2^	Secondary atom site location: difference Fourier map
Least-squares matrix: full	Hydrogen site location: inferred from neighbouring sites
*R*[*F*^2^ > 2σ(*F*^2^)] = 0.061	H-atom parameters constrained
*wR*(*F*^2^) = 0.136	*w* = 1/[σ^2^(*F*_o_^2^) + (0.048*P*)^2^] where *P* = (*F*_o_^2^ + 2*F*_c_^2^)/3
*S* = 1.03	(Δ/σ)_max_ < 0.001
1201 reflections	Δρ_max_ = 0.15 e Å^−^^3^
155 parameters	Δρ_min_ = −0.18 e Å^−^^3^
1 restraint	Extinction correction: none
Primary atom site location: structure-invariant direct methods	

### Special details

**Table:**

Geometry. All esds (except the esd in the dihedral angle between two l.s. planes) are estimated using the full covariance matrix. The cell esds are taken into account individually in the estimation of esds in distances, angles and torsion angles; correlations between esds in cell parameters are only used when they are defined by crystal symmetry. An approximate (isotropic) treatment of cell esds is used for estimating esds involving l.s. planes.
Refinement. Refinement of *F*^2^ against ALL reflections. The weighted *R*-factor *wR* and goodness of fit *S* are based on *F*^2^, conventional *R*-factors *R* are based on *F*, with *F* set to zero for negative *F*^2^. The threshold expression of *F*^2^ > σ(*F*^2^) is used only for calculating *R*-factors(gt) *etc*. and is not relevant to the choice of reflections for refinement. *R*-factors based on *F*^2^ are statistically about twice as large as those based on *F*, and *R*- factors based on ALL data will be even larger.

### Fractional atomic coordinates and isotropic or equivalent isotropic displacement parameters (Å^2^)

**Table:**

	*x*	*y*	*z*	*U*_iso_*/*U*_eq_	
C1	−0.0060 (10)	0.5064 (3)	0.8304 (7)	0.0474 (15)	
C2	−0.0982 (11)	0.5462 (4)	0.6745 (9)	0.0580 (16)	
C3	−0.2999 (13)	0.6076 (4)	0.6777 (10)	0.076 (2)	
H3	−0.3594	0.6343	0.5747	0.091*	
C4	−0.4118 (14)	0.6293 (4)	0.8306 (12)	0.0747 (19)	
H4	−0.5453	0.6712	0.8303	0.090*	
C5	−0.3320 (12)	0.5906 (4)	0.9860 (10)	0.073 (2)	
H5	−0.4150	0.6047	1.0887	0.087*	
C6	−0.1256 (11)	0.5303 (4)	0.9858 (8)	0.0639 (16)	
H6	−0.0652	0.5052	1.0904	0.077*	
C7	0.2044 (10)	0.4416 (3)	0.8361 (7)	0.0480 (14)	
H7	0.2592	0.4179	0.9431	0.058*	
C8	0.5180 (10)	0.3518 (3)	0.7054 (6)	0.0431 (14)	
C9	0.6040 (10)	0.3216 (3)	0.5473 (7)	0.0526 (15)	
C10	0.7977 (11)	0.2575 (4)	0.5400 (8)	0.0657 (17)	
H10	0.8484	0.2375	0.4326	0.079*	
C11	0.9127 (13)	0.2241 (4)	0.6912 (9)	0.0685 (18)	
H11	1.0417	0.1812	0.6877	0.082*	
C12	0.8365 (11)	0.2546 (4)	0.8477 (9)	0.0623 (17)	
H12	0.9182	0.2324	0.9503	0.075*	
C13	0.6393 (11)	0.3180 (3)	0.8582 (7)	0.0565 (15)	
H13	0.5899	0.3373	0.9664	0.068*	
C14	0.4758 (15)	0.3547 (5)	0.3888 (9)	0.093 (2)	
N1	0.3182 (9)	0.4157 (2)	0.6972 (5)	0.0459 (11)	
N2	0.3736 (15)	0.3796 (5)	0.2608 (8)	0.147 (3)	
O1	0.0028 (9)	0.5256 (3)	0.5203 (5)	0.0815 (14)	
H1	0.1174	0.4881	0.5348	0.122*	

### Atomic displacement parameters (Å^2^)

**Table:**

	*U*^11^	*U*^22^	*U*^33^	*U*^12^	*U*^13^	*U*^23^
C1	0.040 (3)	0.047 (4)	0.055 (4)	−0.003 (3)	0.005 (3)	−0.002 (3)
C2	0.052 (3)	0.044 (4)	0.079 (5)	0.005 (3)	0.013 (3)	0.011 (3)
C3	0.067 (5)	0.068 (5)	0.094 (6)	0.010 (4)	0.014 (4)	0.020 (4)
C4	0.063 (4)	0.043 (4)	0.119 (6)	0.012 (4)	0.013 (4)	−0.001 (4)
C5	0.051 (4)	0.079 (5)	0.089 (5)	0.002 (3)	0.014 (4)	−0.029 (4)
C6	0.054 (4)	0.075 (5)	0.062 (4)	−0.004 (4)	−0.002 (3)	−0.020 (3)
C7	0.043 (3)	0.053 (4)	0.047 (4)	0.002 (3)	−0.003 (2)	−0.005 (3)
C8	0.046 (3)	0.042 (4)	0.042 (3)	−0.002 (3)	0.004 (2)	−0.006 (2)
C9	0.050 (3)	0.060 (4)	0.048 (4)	0.004 (3)	0.001 (3)	0.003 (3)
C10	0.065 (4)	0.068 (5)	0.065 (4)	0.009 (3)	0.005 (3)	−0.017 (3)
C11	0.060 (4)	0.072 (5)	0.073 (5)	0.007 (4)	0.004 (3)	−0.005 (4)
C12	0.056 (4)	0.052 (4)	0.080 (5)	0.010 (3)	0.006 (3)	0.017 (3)
C13	0.059 (4)	0.061 (4)	0.050 (4)	0.006 (3)	0.010 (3)	0.007 (3)
C14	0.093 (5)	0.135 (7)	0.052 (4)	0.042 (5)	0.005 (4)	−0.012 (4)
N1	0.049 (3)	0.041 (3)	0.048 (3)	−0.003 (2)	0.0079 (19)	0.000 (2)
N2	0.162 (7)	0.225 (9)	0.053 (4)	0.106 (6)	−0.002 (4)	0.010 (5)
O1	0.085 (3)	0.092 (4)	0.069 (3)	0.028 (2)	0.020 (2)	0.030 (2)

### Geometric parameters (Å, °)

**Table:**

C1—C6	1.405 (7)	C8—C13	1.389 (7)
C1—C2	1.406 (7)	C8—C9	1.391 (6)
C1—C7	1.450 (6)	C8—N1	1.406 (6)
C2—O1	1.345 (7)	C9—C10	1.393 (7)
C2—C3	1.385 (8)	C9—C14	1.433 (9)
C3—C4	1.363 (9)	C10—C11	1.365 (8)
C3—H3	0.9300	C10—H10	0.9300
C4—C5	1.381 (9)	C11—C12	1.366 (8)
C4—H4	0.9300	C11—H11	0.9300
C5—C6	1.387 (8)	C12—C13	1.397 (7)
C5—H5	0.9300	C12—H12	0.9300
C6—H6	0.9300	C13—H13	0.9300
C7—N1	1.293 (5)	C14—N2	1.144 (7)
C7—H7	0.9300	O1—H1	0.8200
			
C6—C1—C2	118.3 (5)	C13—C8—C9	117.9 (5)
C6—C1—C7	119.1 (5)	C13—C8—N1	125.2 (5)
C2—C1—C7	122.6 (5)	C9—C8—N1	116.9 (4)
O1—C2—C3	118.5 (6)	C8—C9—C10	121.8 (5)
O1—C2—C1	121.7 (5)	C8—C9—C14	118.4 (5)
C3—C2—C1	119.8 (6)	C10—C9—C14	119.7 (5)
C4—C3—C2	120.5 (6)	C11—C10—C9	119.7 (6)
C4—C3—H3	119.8	C11—C10—H10	120.2
C2—C3—H3	119.8	C9—C10—H10	120.2
C3—C4—C5	121.6 (6)	C10—C11—C12	119.3 (6)
C3—C4—H4	119.2	C10—C11—H11	120.3
C5—C4—H4	119.2	C12—C11—H11	120.3
C4—C5—C6	118.6 (6)	C11—C12—C13	122.0 (6)
C4—C5—H5	120.7	C11—C12—H12	119.0
C6—C5—H5	120.7	C13—C12—H12	119.0
C5—C6—C1	121.2 (6)	C8—C13—C12	119.3 (5)
C5—C6—H6	119.4	C8—C13—H13	120.3
C1—C6—H6	119.4	C12—C13—H13	120.3
N1—C7—C1	122.1 (4)	N2—C14—C9	178.6 (8)
N1—C7—H7	118.9	C7—N1—C8	121.1 (4)
C1—C7—H7	118.9	C2—O1—H1	109.5

### Hydrogen-bond geometry (Å, °)

**Table:**

*D*—H···*A*	*D*—H	H···*A*	*D*···*A*	*D*—H···*A*
O1—H1···N1	0.82	1.92	2.651 (6)	147

## References

[bb1] Allen, F. H., Kennard, O., Watson, D. G., Brammer, L., Orpen, A. G. & Taylor, R. (1987). *J. Chem. Soc. Perkin Trans. 2*, pp. S1–19.

[bb2] Burnett, M. N. & Johnson, C. K. (1996). *ORTEPIII* Report ORNL-6895. Oak Ridge National Laboratory, Oak Ridge, Tennessee, USA.

[bb3] Chen, Z. H., Morimoto, H., Matsunaga, S. & Shibasaki, M. (2008). *J. Am. Chem. Soc.* **130**, 2170–2171. 10.1021/ja710398q 18225906

[bb4] Cheng, K., You, Z.-L., Li, Y.-G. & Zhu, H.-L. (2005). *Acta Cryst.* E**61**, o1137–o1138.

[bb5] Cheng, K., Zhu, H.-L., Li, Z.-B. & Yan, Z. (2006). *Acta Cryst.* E**62**, o2417–o2418.

[bb6] Elmah, A., Kabak, M. & Elerman, Y. (1999). *J. Mol. Struct.* **484**, 229–234.

[bb7] Farrugia, L. J. (1997). *J. Appl. Cryst.* **30**, 565.

[bb8] May, J. P., Ting, R., Lermer, L., Thomas, J. M., Roupioz, Y. & Perrin, D. M. (2004). *J. Am. Chem. Soc.* **126**, 4145–4156. 10.1021/ja037625s 15053604

[bb9] Rigaku (2005). *CrystalClear* Rigaku Corporation, Tokyo, Japan.

[bb10] Sheldrick, G. M. (2008). *Acta Cryst.* A**64**, 112–122. 10.1107/S0108767307043930 18156677

[bb11] Weber, B., Tandon, R. & Himsl, D. (2007). *Z. Anorg. Allg. Chem.* **633**, 1159–1162.

[bb12] Xu, H.-J., Gong, X.-X. & Wang, H. (2008). *Acta Cryst.* E**64**, o638. 10.1107/S1600536808005242 PMC2960747 21201969

